# Different techniques for caudal extension graft placement in rhinoplasty^?^

**DOI:** 10.1016/j.bjorl.2019.08.002

**Published:** 2019-09-14

**Authors:** Amir Arvin Sazgar, Azadeh Kheradmand, Ali Razfar, Shabnam Hajialipour, Amir Keyvan Sazgar

**Affiliations:** a Tehran University of Medical Sciences, Vali-Asr Hospital, Department of Otolaryngology Head and Neck Surgery, Tehran, Iran; b University of California, Los Angeles Medical Center, Department of Otolaryngology Head and Neck Surgery, Los Angeles, United States; c Tabriz University of Medical Sciences, Imam Reza Hospital, Department of Otolaryngology Head and Neck Surgery, Tehran, Iran

**Keywords:** Septal deviation, Caudal extension graft, Tongue-in-groove technique, Septorhinoplasty

## Abstract

**Introduction:**

The caudal extension graft is usually a cartilage graft that overlaps the caudal margin of the nasal septum. A combination of the caudal extension graft and the tongue-in-groove technique is used to stabilize the nasal base, set tip projection, and refine the alar-columellar relationship.

**Objectives:**

In this study we present some new modifications to the placement of caudal extension grafts in rhinoplasty.

**Methods:**

This study is a retrospective review of a prospectively collected database of 965 patients who underwent septorhinoplasty from June 2011 to July 2015. Of these, 457 patients required a caudal extension graft and were included in the study. Minimum follow-up was 13.2 months with a mean follow-up time of 17.4 months.

**Results:**

In most cases, comparison of photographs before and after surgery were satisfactory and showed improved contour. Minor deformity was detected in 41 patients and 11 patients needed revision surgery.

**Conclusion:**

With these modifications the surgeon can employ the caudal extension graft even in angulated caudal septal deviations. A variety of methods have been proposed for correction of caudal nasal deviation.

## Resumo

**Introdução:**

O enxerto de extensão caudal é geralmente um enxerto de cartilagem que se sobrepõe à margem caudal do septo nasal. Uma combinação do enxerto de extensão caudal e a técnica de *tongue-in-groove* é utilizada para estabilizar a base nasal, definir a projeção da ponta e refinar a relação alar-columelar.

**Objetivos:**

Neste estudo apresentamos algumas novas modificações na colocação dos enxertos de extensão caudal na rinoplastia.

**Método:**

Este estudo é uma revisão retrospectiva de um banco de dados prospectivamente coletado de 965 pacientes que se submeteram a rinosseptoplastia de junho de 2011 a julho de 2015. Destes, 457 pacientes necessitaram de enxerto de extensão caudal e foram incluídos no estudo. O seguimento mínimo foi de 13,2 meses, com tempo médio de seguimento de 17,4 meses.

**Resultados:**

Na maioria dos casos, a comparação das fotografias antes e após a cirurgia foi satisfatória e apresentou melhora do contorno. Pequenas deformidades foram detectadas em 41 pacientes e 11 pacientes necessitaram de cirurgia de revisão.

**Conclusão:**

Com essas modificações, o cirurgião pode utilizar o enxerto de extensão caudal mesmo em desvios angulares do septo caudal. Vários métodos têm sido propostos para correção do desvio septo-caudal.

## Introduction

The caudal extension graft (CEG) is normally a cartilage graft that overlaps the caudal margin of the nasal septum and is sutured between the medial crura of the alar cartilages. A combination of the CEG and the Tongue-In-Groove (TIG) technique is used to stabilize the nasal base, set tip projection, and refine the alar-columellar relationship.^1^ The position of CEG will determine the position of the nasal tip, nostril shape, columellar thickness and nasolabial angle. These techniques produce good tip support, however, if the septum is deviated and the tip displaced, the shape and condition of the tip is related to the septum.^2^, ^3^

Straightening a deviated caudal septum is the most critical component in successful correction of a deviated tip.^4^ Correction of a severely deviated or deformed caudal septum during septoplasty can be a particularly challenging task because the septum plays a primary role in the ultimate appearance of the nose.

A variety of methods have been proposed for correction of caudal nasal deviation including: conventional septoplasty, cartilage “plating” rigid fixation technique,^5^ septal crossbar graft,^6^ septal batten techniques,^7^ asymmetric spreader graft techniques, extracorporeal septoplasty, two-dimensional L-strut reconstruction^8^ and CEG with TIG.^1^, ^2^, ^3^ Recently some suture techniques have also introduced for correction of caudal nasal deviation.^9^, ^10^, ^11^ However, many of these methods were not used in combination with TIG techniques. Generally, surgeons believe that placement of the CEG next to the septum that is slightly off the midline is easier than placement of the CEG next to the septum that is exactly in the midline. In this article we describe our techniques for placement of the CEG in the correction and stabilization of caudal septal deformities and evaluate the effectiveness of these various techniques.

## Methods

This retrospective study was conducted in both university and private practice settings. The institutional review board approved the current method (the ethical code: IR.TUMS.VCR.REC.1395.1141), and each patient gave informed consent for the procedure. Patients with prior history of rhinoplasty, septoplasty, or any kind of congenital nasal anomaly, including cleft nose deformity, and patients that required extracorporeal or different types of modified extracorporeal septoplasty were excluded from the study.

All surgical operations were performed by one surgeon (AAS), under general anesthesia with additional infiltration of local anesthetics. An open approach via trans columellar and bilateral marginal incisions was used for all patients. The skin was dissected and raised in the supraperichondrial and subperiosteal planes over the nasion. Through a standard open rhinoplasty approach, the nasal septum was exposed via mucoperichondrial and mucoperiosteal flaps. If cartilage grafts were required or the cartilage was deviated or deformed, septal cartilage was resected as described below.

## Surgical techniques

### Septal extension graft

In cases having slight caudal deviation with caudal retraction, a septal extension graft can be used. In this scenario the L-strut of the septum was preserved. A majority of patients are in this group (423 patients in this research).

### Sliding septum

**Figure 1:**
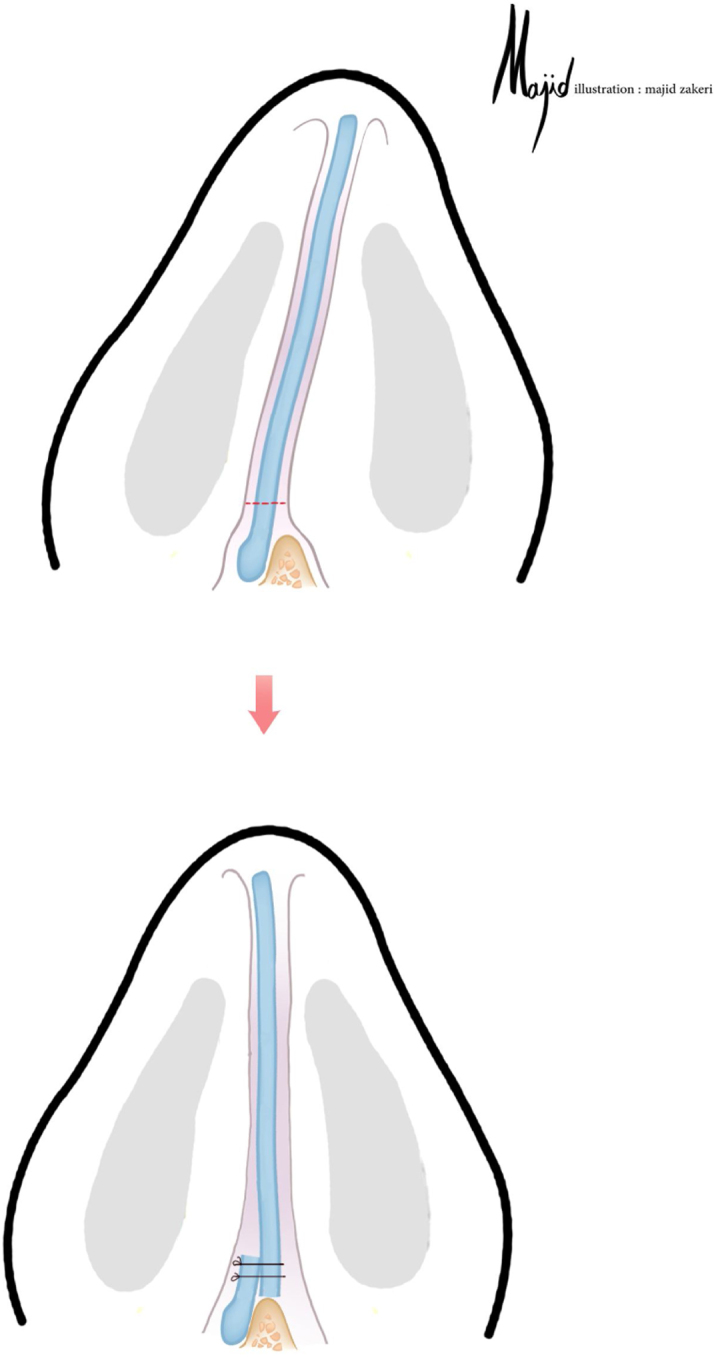
When the caudal part of the L-strut is tilted the sliding technique can be used. The caudal extension graft is usually placed on the opposite side from the deviation (Illustration by Majid Zakeri).

This technique is used in cases with a straight but tilted caudal septal deviation. The most deviated central inferior portion of the septal cartilage is resected, preserving the cartilaginous L-strut. Then, the caudal septal part is incised leaving 3?4 mm of cartilage attached to the Anterior Nasal Spine (ANS). The septum is then transferred to the opposite side of the deviation and secured to the remnant cartilage overlying the ANS. Subsequently, the extension graft is placed next to the septum (Fig. 1). 14 patients were included in this technique..

### Bar graft with caudal extension graft

**Figure 2:**
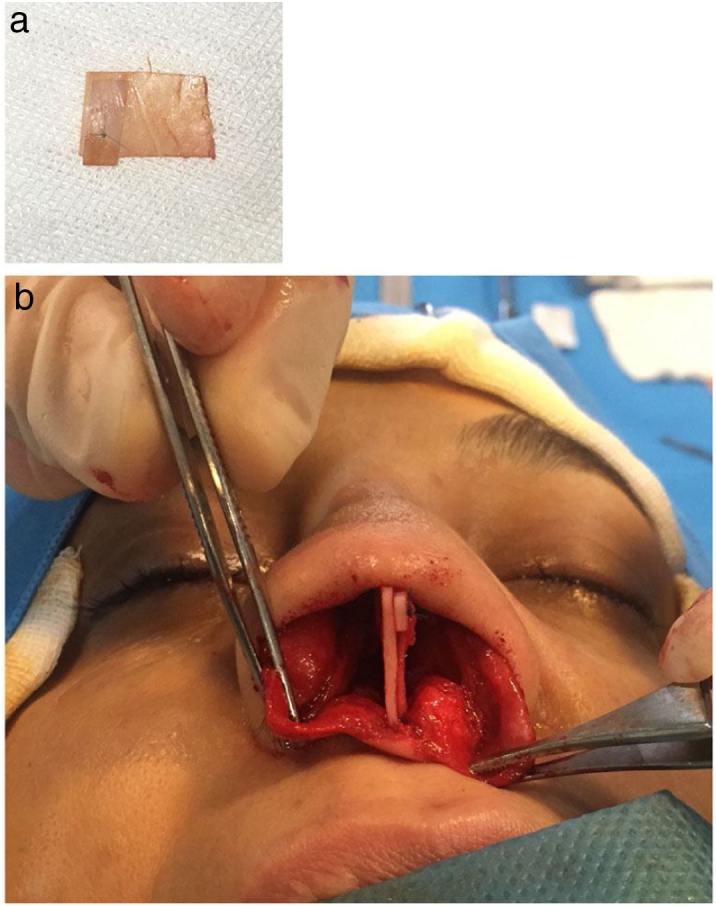
(a) Prefabricated caudal extension graft made by suturing a bar graft on it; (b) The bar graft is placed on the left side of the caudal septum and fixed in the empty space between the septum and caudal graft.

This technique is used in cases with linear septal deviation lateral to the ANS without retraction. A strip of cartilage with sufficient height and width may be placed at the caudal septal area to correct deviation. A bar graft is placed and fixed in the empty space between the caudal septum and caudal graft. This is useful in cases where the native septum cannot be straightened completely. Thus, the caudal graft provides a strong base that is straight for TIG techniques (Fig. 2). 9 patients were included in this technique..

### Interlaced cartilages

**Figure 3:**
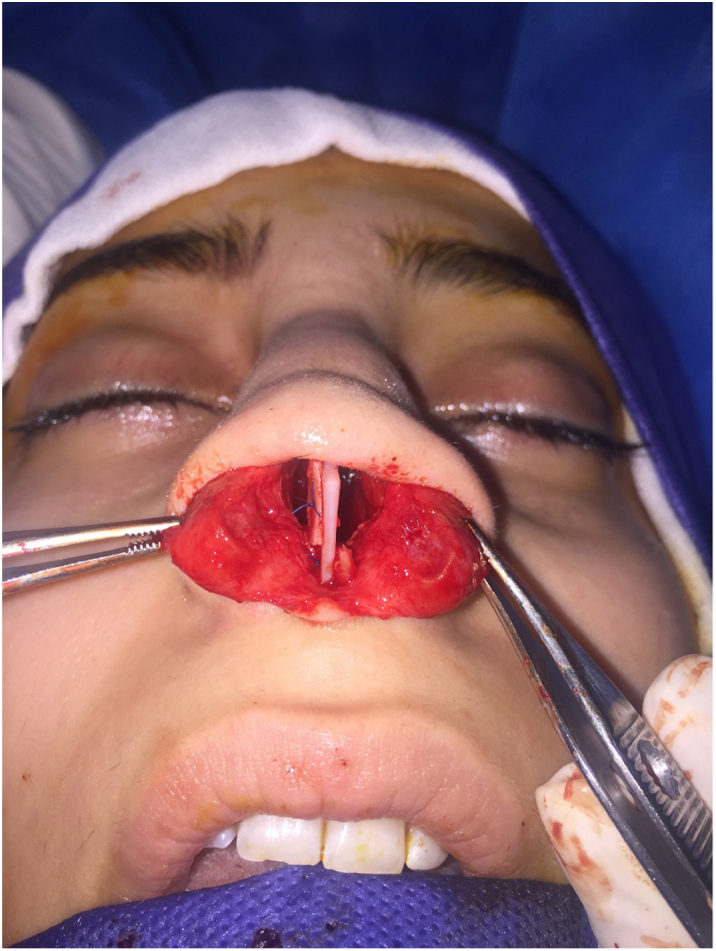
The caudal extension graft was placed between the two parts of the caudal septum in the interlaced cartilage modification. The caudal septum was divided along the site of previous angulation.

This technique is used in cases with an angulated caudal septal cartilage. The septal cartilage is incised in angulation and the septal extension graft is placed between the two pieces. The septal cartilage is fixed alternately in the right and in the left side of CEG, as if woven together (Fig. 3). 11 patients were involved in this group.

In addition, some common surgical procedures were used for all patients, including auto spreader flap placement, tongue-in-grove technique and suture technique tip plasty. Bony and cartilaginous hump removal as well as medial and lateral osteotomy and alar base resection were performed as needed. Other types of tip grafts were placed rarely. Nasal packing was not used for any of the patients.

## Result

This study is a retrospective review of a prospectively collected database of 965 patients (227 males and 738 females) who underwent septorhinoplasty between June 30, 2011 and July 1, 2015. Patient ages ranged from 16 to 65 (mean = 31.3). Minimum follow-up was 13.2 months with a mean follow-up of 17.4 months. In the present study, 457 patients required caudal extension grafts. Of these, corrections were made on 423 patients with simple placement of caudal extension grafts (314 on the right side and 109 on the left side), 14 patients with the sliding method, 9 patients with bar grafts and 11 patients with the interlaced method. Different maneuvers during the operation were shown in the table (Table 1).

**Table 1:** Summary of intraoperation maneuvers.

Operative technique	n = 457
*Bony hump removal*	
Less than 2 mm^a^	229 (50.1%)
2 to 4 mm	143 (31.2%)
More than 4 mm	16 (3.5%)

*Cartilaginous hump removal*	
Less than 2 mm^a^	210 (45.9%)
2 to 4 mm	140 (30.6%)
More than 4 mm	20 (4.3%)

*Cephalic portion of lateral crus*	
Hing flap was made^b^	319 (69.3%)
Trimmed	113 (24.7%)

*Tip sutures*	
Trans domal	402 (87.9%)
Inter domal	400 (87.5%)
Lateral crural spanning	127 (27.7%)

*Osteotomy*	
Transvers medial	427 (93%)
Lateral	436 (95.6%)

a The patients needed dorsal augmentation was not included.

b Hinge flap is a technique that the LLC and ULC were not separated in the scroll area.^16^

**Figure 4:**
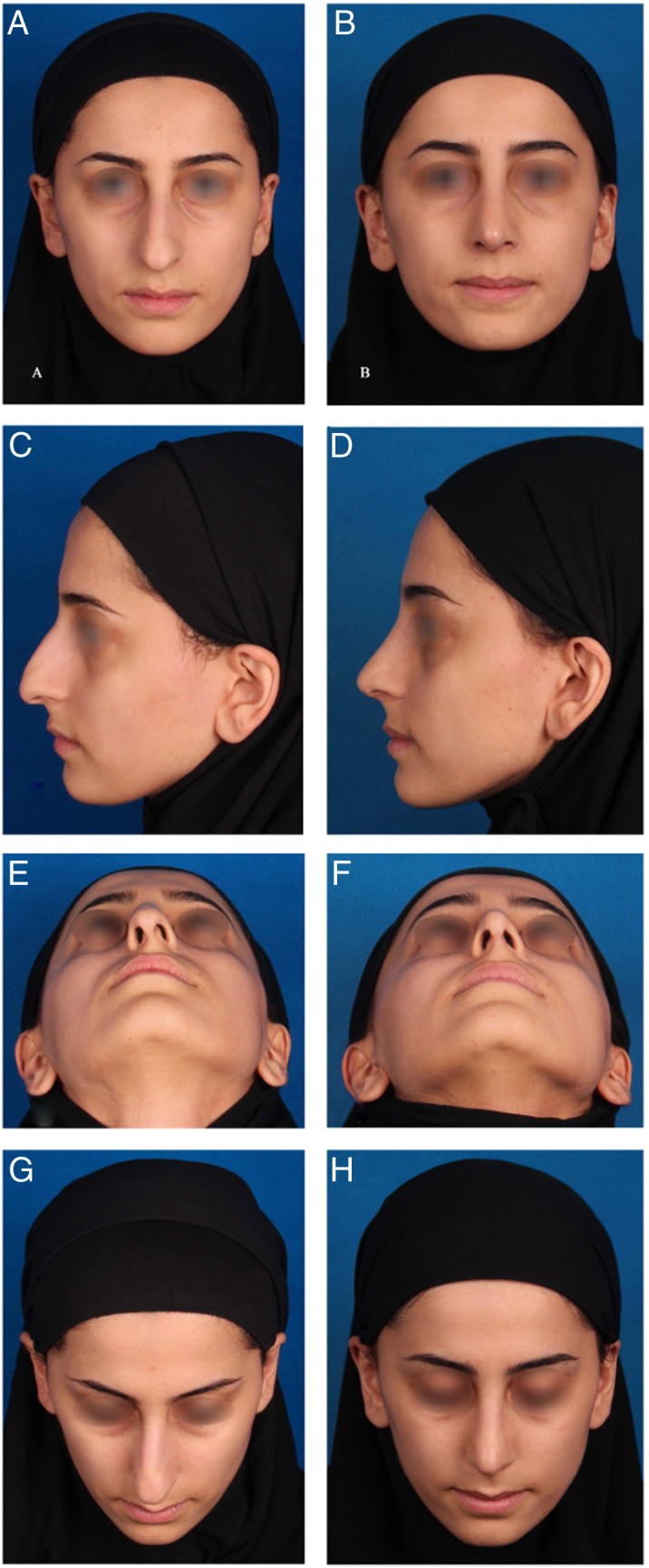
(A, C, E, G) Preoperative and (B, D, F, H) 11 month postoperative views of a 25 year-old woman who underwent cosmetic rhinoplasty with Interlaced cartilages technique. The main problem was tension nose with sever deviation specially in basal view. In this case the bony and cartilaginous hump removal, bilateral auto spreader flaps, medial-transvers and lateral osteotomy and suture technique tip plasty was also performed.

In all cases, the surgeon compared pre- and post-operative photography (Fig. 4). Minor deformity was detected in 41 patients and 11 patients needed revision surgery. Minor deformity was noticed in 37 patients of the septal extension graft group, in one patient of sliding technique group, in two patients of bar graft with caudal extension graft group and in one patient of interlaced cartilages technique group. Revision surgery was needed in 10 patients of the septal extension graft group and in one patient of the bar graft with caudal extension graft group. Postoperative complications occurred in five patients. Two patients had postoperative hemorrhaging, one patient experienced axillary vein thrombosis, one patient had a nasal abscess and one developed synechia.

## Discussion

Correction of caudal septal deformities is a critical step in septorhinoplasty. Identification of the exact septal deformity is paramount in appropriate selection of surgical technique. Metzenbaum was one of the first to describe a procedure for the correction of the caudal septum.^12^ He introduced the “swinging door” technique where the convex portion of the caudal septum was resected to move the septum to the midline. Modifications of this technique have been introduced to correct caudal septal deformities with suture fixation to the ANS.^13^, ^14^ These techniques are only effective in cases where the septum is straight but “tilted” to one side. The present research utilized two different techniques in this particular deformity: the sliding technique and the bar graft with the CEG technique. The location of the ANS is an important anatomical consideration in determining which technique is more effective. In patients with an ANS that is off midline in conjunction with caudal septal tilting, the sliding technique can be utilized. In patients with a septal tilting that is positioned on a midline ANS, the bar technique is effective. It must be noted that there is a slight loss of projection with the sliding technique, which must be compensated.

Patients with curved deformities of the caudal septum comprise a unique dilemma. Evaluating both dorsal and caudal contributions to the overall deformity is critical in selecting appropriate techniques. For patients with mild C-shaped deformities of the caudal septum, cartilaginous septal extension grafts are effective. However, patients with severe C-shaped or S-shaped deformities involving the dorsal strut require a much more aggressive approach. A composite “spreader” (bone from vomer) can be used, resulting less enlargement of the nasal tip. However, extracorporeal septoplasty is a mainstay in the treatment of this challenging septum. In a large cohort of patients, Gubisch illustrated the effectiveness of this technique in correction of marked deviated septum. However, this technique did result in saddling and dorsal irregularities in 8% of patients.^15^ In some patients with angulated cartilage, especially in cases with caudal deviation without dorsal deviation, the extension graft can be placed using the interlaced method.

Effective treatment of caudal septal deformities, including cartilage deficiency and deviation, significantly improves both functional and aesthetic outcomes in rhinoplasty. Unrepaired caudal deformity can cause twisting of the lower third of the nose affecting both tip position and symmetry.^2^ The correction of caudal deviations in patients with cartilaginous deficiency or those with small septal cartilages creates an added level of complexity. The CEG has proven to be a versatile graft in these patients. This graft is particularly useful in patients with columellar retraction and poor tip projection.

## Conclusion

With the popularity of using CEG and TIG, various techniques were introduced to match the CEG placement for different scenarios. This research shows that with these modifications the surgeon can use CEG even in deviated or angulated caudal septa. This study is a retrospective study and some comparative studies are needed to clarify the result.

## Conflicts of interest

The authors declare no conflicts of interest.
